# Senolytic elimination of senescent macrophages restores muscle stem cell function in severely dystrophic muscle

**DOI:** 10.18632/aging.204275

**Published:** 2022-09-08

**Authors:** Lei Liu, Xianlin Yue, Zewei Sun, William S. Hambright, Qi Feng, Yan Cui, Johnny Huard, Paul D. Robbins, Zhihui Wang, Xiaodong Mu

**Affiliations:** 1 Shandong The First Medical University and Shandong Academy of Medical Sciences, Jinan, Shandong, China; 2 Center for Regenerative Sports Medicine, Steadman Philippon Research Institute, Vail, CO 81657, USA; 3 University of Texas Health Science Center at Houston, Houston, TX 77030, USA; 4 Department of Biochemistry, Molecular Biology and Biophysics, Institute on the Biology of Aging and Metabolism, University of Minnesota, Minneapolis, MN 55455, USA

**Keywords:** muscular dystrophy, senolytics, cellular senescence, immunosenescence, stem cells

## Abstract

The aging of the immune system, or immunosenescence, was recently verified to have a causal role in driving the aging of solid organs, while the senolytic elimination of senescent immune cells was found to effectively delay systemic aging. Our recent study also showed that immune cells in severely dystrophic muscles develop senescence-like phenotypes, including the increased expression of senescence-associated secretory phenotype (SASP) factors and senescence markers. Here we further investigated whether the specific clearance of senescent immune cells in dystrophic muscle may effectively improve the function of muscle stem cells and the phenotypes of dystrophic muscle. We observed increased percentage of senescent cells in macrophages from *mdx*/utro(−/−) mice (a murine model for muscular dystrophy disease, dystrophin−/−; utrophin−/−), while the treatment of *mdx*/utro(−/−) macrophages with senolytic drug fisetin resulted in reduced number of senescent cells. We administrated fisetin to *mdx*/utro(−/−) mice for 4 weeks, and observed obviously reduced number of senescent immune cells, restored number of muscle cells, and improve muscle phenotypes. In conclusion, our results reveal that senescent immune cells, such as macrophages, are greatly involved in the development of muscle dystrophy by impacting the function of muscle stem cells, and the senolytic ablation of these senescent cells with fisetin can be an effective therapeutic strategy for improving function of muscle stem cells and phenotypes of dystrophic muscles.

## INTRODUCTION

Duchenne Muscular Dystrophy (DMD) is caused by mutations of the dystrophin gene, which leads to progressive muscle fiber damage and degeneration, resulting in cardiac or respiratory failure and ultimately premature death [1, 2]. DMD patients develop severe chronic inflammation in their muscles, which is characterized by the massive infiltration of immune cells into muscles and elevated activation of pro-inflammatory signaling in muscle cells [1, 2]. Our previous studies of *mdx*/Utro(−/−) mice (a severe murine model for DMD; *dystrophin−/−*; utrophin−/−) revealed the greatly increased number of SA-β-gal (Senescence-associated β-Galactosidase) + senescent cells in the dystrophic muscles, which is closely associated with severe dystrophic phenotypes [3, 4]. Our recent study further revealed that, macrophages in dystrophic muscles have aberrant activation of RhoA signaling, which is associated with upregulated expression of SASP (Senescence-associated secretary phenotypes) factors and senescence-like features of macrophages [3]. Therefore, it would be interesting to find out whether the repression or elimination of these senescence-like macrophages can be effective in recuing dystrophic muscle phenotypes.

One recent important progression in the studying of ageing mechanism has been the observation that the ageing immune system, or immunosenescence, has a causal role in driving systemic ageing and therefore represents a key therapeutic target to delay ageing [5]. It was verified that the senolytic ablation of senescent immune cells in the aged organism can effectively delay the systemic aging [5]. In normal conditions, senescent cells accumulated in body are specifically identified and removed by immune system [6, 7]; however, when the immune cell become senescent too, their ability to clear senescent cells would be greatly compromised, leading to excessive accumulation of senescent cells and aging-related disorders [5–7]. Therefore, potential clearance or repression of senescent immune cells would be greatly beneficial for body health.

Senescent cells accumulate with age and are considered to be greatly related to age-related disorders [5, 8]. Senescence is a cell fate elicited in response to external and internal cellular stress signals, which causes extensive changes in gene expression, histone modifications, organelle function, elevated protein production, and profound morphologic and metabolic shifts [9]. Senescent cells usually actively secret SASP factors, which act on the non-senescent normal cells and interfere with their normal function [10–12]. In recent years, elimination of senescent cells with senolytic drugs (senolytics) have arisen as a novel anti-aging strategy and therapeutic approach for treating many age-related diseases [13–20]. Senolytics are natural or synthesized compounds, including dasatinib and quercetin (D+Q), navitoclax (ABT263), 17-DMAG, and fisetin [13–16, 21]. Senolytics can selectively eliminate senescent cells by targeting anti-apoptotic signaling pathways that are overtly upregulated in senescent cells, such as BCL2 and BCL-XL [13–16, 21].

Therefore, in the current study, we investigated whether the treatment of utrophin−/−mdx mice with senolytic drug (fisetin) can be effective in eliminating the senescent immune cells and improve muscle phenotypes. We also performed *in vitro* treatment of cell co-culture system of immune cells and muscle stem cells to verify our observation *in vivo*. Our results will help verify the potential causal role of senescent immune cells in leading to severe muscle dystrophy and present a novel strategy for therapeutic treatment of DMD disease with senolytic drugs.

## RESULTS

### Skeletal muscle of *mdx*/utro(−/−) mice develops higher level of muscle stem cell exhaustion, immune cell infiltration, and cellular senescence

**Figure 1:**
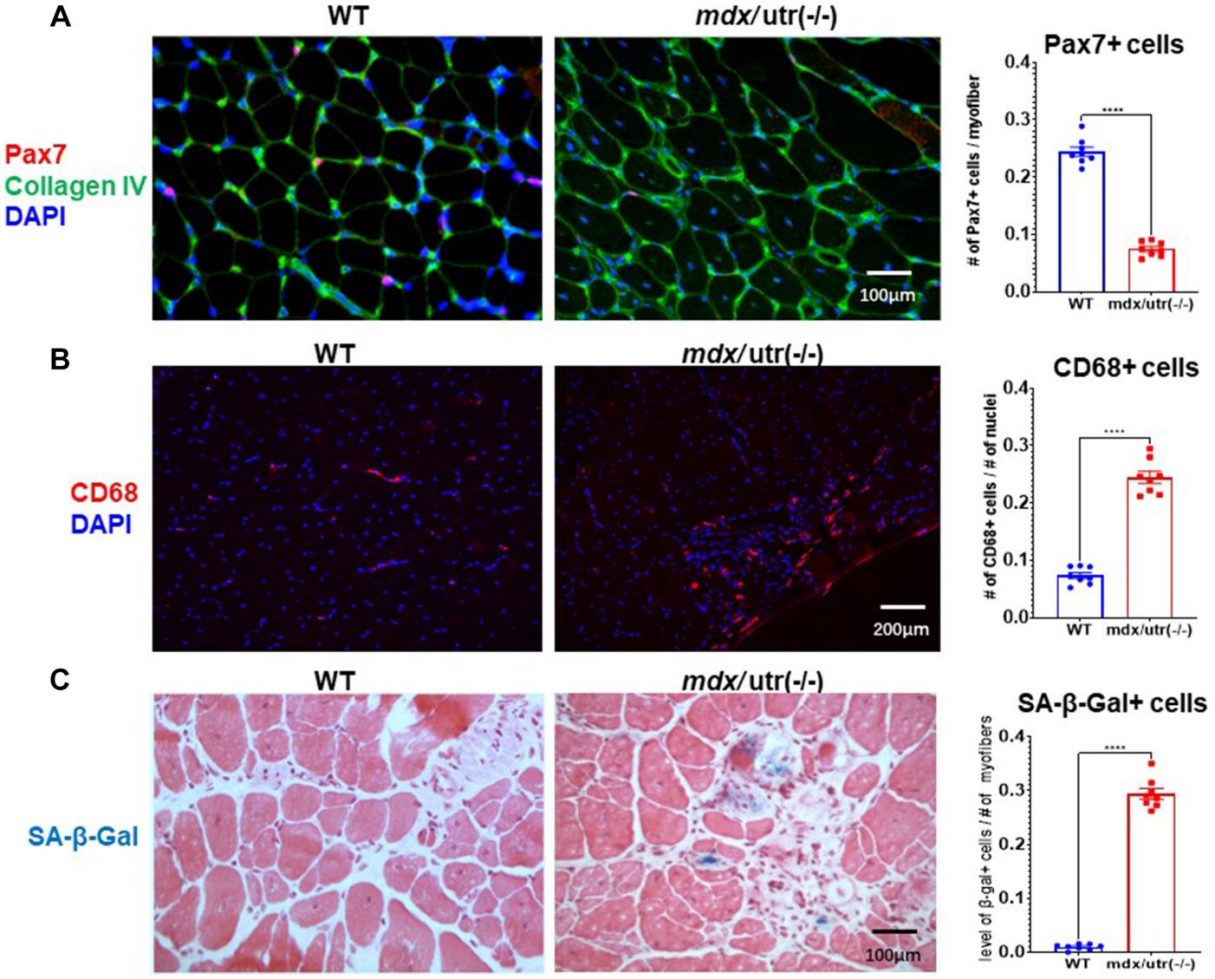
***mdx*/utro(−/−) muscle exhibits obvious stem cell exhaustion, immune cell infiltration and cellular senescence.** (**A**) Immunofluorescent staining of Pax7 and Collagen type IV in muscle tissues of WT and *mdx*/utro(−/−) mice. (**B**) Immunofluorescent staining of CD68 in muscle tissues of WT and *mdx*/utro(−/−) mice. (**C**) Staining of SA-β-Gal in muscle tissues of WT and *mdx*/utro(−/−) mice. *n* = 8 mice/group for statistics, and data are presented as mean +/− SD.

Gastrocnemius muscles from hindlimbs were collected from 8-week old WT and *mdx*/utro(−/−) mice for histological analyses (Figure 1). Immunofluorescent staining of Pax7 and collagen type IV in muscle tissues revealed that, there is significantly reduced number of Pax7+ muscle stem cells in *mdx*/utro(−/−) muscles (Figure 1A); while in contrast, immunofluorescent staining of CD68 showed that, the number of CD68+ macrophages is greatly increased in *mdx*/utro(−/−) muscles (Figure 1B). Meanwhile, SA-β-Gal (senescence-associated β-Galactosidase, a marker for cellular senescence) staining further revealed that, there is obviously higher number of SA-β-Gal+ senescent cells in *mdx*/utro(−/−) muscles (Figure 1C).

### CD68+ macrophages in *mdx*/Utro(−/−) muscle features higher level of mTORC1 activation

**Figure 2:**
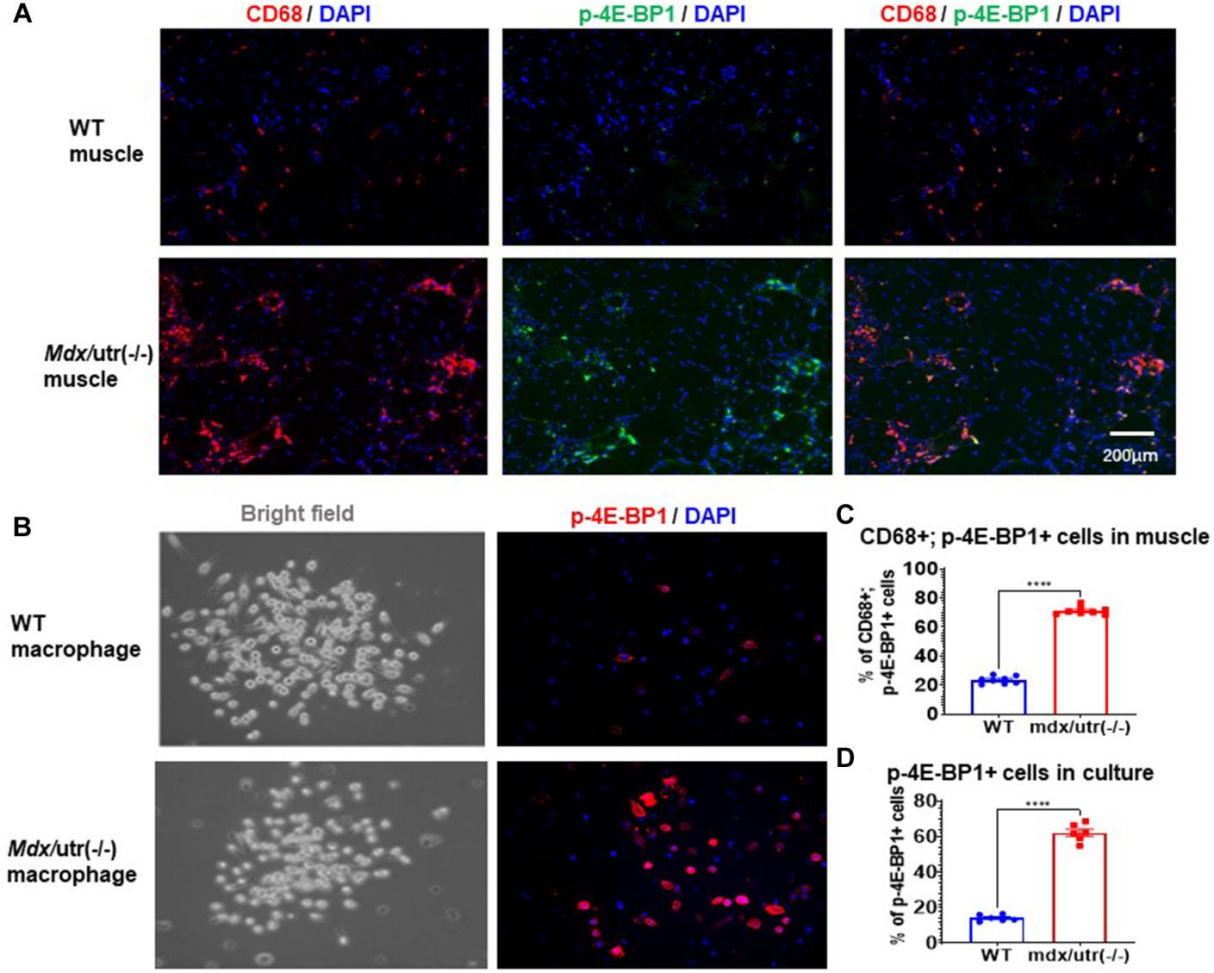
**Macrophages in *mdx*/utro(−/−) muscle exhibits higher level of mTOR activation.** (**A**) Immunofluorescent staining of CD68 and p-4E-BP1 in muscle tissues of WT and *mdx*/utro(−/−) mice. (**B**) Immunofluorescent staining of p-4E-BP1 in macrophages isolated from muscle tissues of WT and *mdx*/utro(−/−) mice. (**C**) Statistics of results *in vivo*. *n* = 8 mice/group. Data are presented as mean +/− SD. (**D**) Statistics of result *in vitro*, *n* = 6 (3 cell lineages x 2 replicates/group). Data are presented as mean +/− SD.

Our previous study of *mdx*/utro(−/−) mice revealed that, CD68+ macrophages in dystrophic muscle develop senescence-like phenotypes, including higher level of positive C_12_FDG (a fluorogenic substrate for β-galactosidase) signaling [3]. mTORC1 activation has been known as the central regulator of cellular senescence, which occurs in senescent human cells independent of growth factor signaling [22, 23]. In order to further verify whether CD68+ macrophages in *mdx*/utro(−/−) muscle may develop higher level of cellular senescence, co-immunostaining of CD68 and p-4E-BP1 (a substrate for mTORC1 signaling) in muscle tissues was performed. Results showed that, CD68+ macrophages in *mdx*/utro(−/−) muscle express higher amount of p-4E-BP1 protein (Figure 2A, 2C), indicating a high level of mTOR activation and senescence.

Also, CD68+ macrophages were isolated from WT and *mdx*/utro(−/−) muscles via flow cytometry based on CD68 expression, to compare their expression of p-4E-BP1 protein, and we observed significantly higher percentage of p-4E-BP1+ cells in CD68+ macrophages from *mdx*/utro(−/−) muscle (Figure 2B, 2D).

### CD68+ macrophages from *mdx*/Utro(−/−) muscle features elevated expression of SASP factors and increased cellular senescence

**Figure 3:**
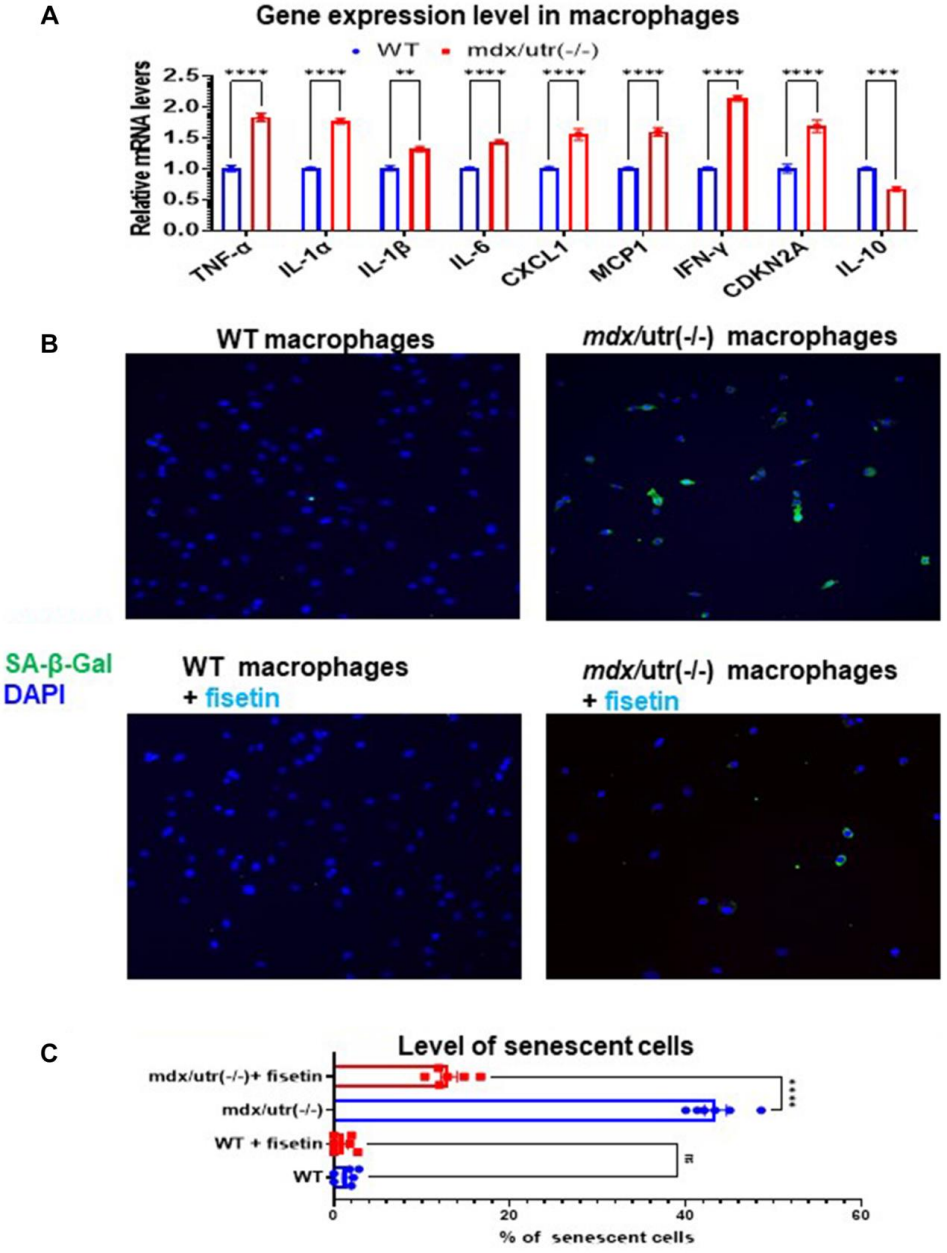
**Fisetin treatment specifically remove senescent macrophages from *mdx*/utro(−/−) mouse.** (**A**) Real time-PCR result of gene expression level of SASP factors, p16 and IL-10 in WT and *mdx*/utro(−/−) macrophages (*n* = 3 cell lineages). (**B**) Staining of SA-β-Gal in 4 groups of macrophages with fluorescent Senescence Assay Kit. Fisetin was applied to treat the WT and *mdx*/utro(−/−) macrophages for 48 hr at 20 μM of concentration. (**C**) Statistics of SA-β-Gal+ cells in 4 groups of cells. *n* = 6 (3 cell lineages x 2 replicates/group). Data are presented as mean +/− SD.

The higher activation of mTOR signaling in *mdx*/utro(−/−) macrophage may suggest a potential development of cellular senescence. Thus, we then compared the expression of SASP factors in CD68+ macrophages isolated from WT and *mdx*/utro(−/−) muscles. RT-PCR result showed that, CD68+ macrophages from *mdx*/utro(−/−) muscle displayed up-regulated expression of SASP factors (i.e., TNF-α, IL-1α, IL-1β, IL-6, CXCL1, MCP1, and IFN-γ) and p16, and down-regulated expression of anti-inflammation factor IL10 (Figure 3A). Meanwhile, the fluorescent staining of SA-β-Gal revealed that there was higher percentage of SA-β-Gal+ cells in *mdx*/utro(−/−) macrophages, compared with WT macrophages (Figure 3B, 3C).

Further, we examined whether the senolytic drug fisetin may specifically remove SA-β-Gal+ senescent cells in *mdx*/utro(−/−) macrophages. Fisetin was applied to treat the WT and *mdx*/utro(−/−) macrophages at 20 μM of concentration for 48 hr. Result showed that, the percentage of SA-β-Gal+ senescent cells in *mdx*/utro(−/−) macrophages was obviously reduced after fisetin treatment (Figure 3B, 3C).

### CD68+ macrophages isolated from *mdx*/utro(−/−) muscle negatively impact the function of muscle stem cell in a cell co-culture system

**Figure 4:**
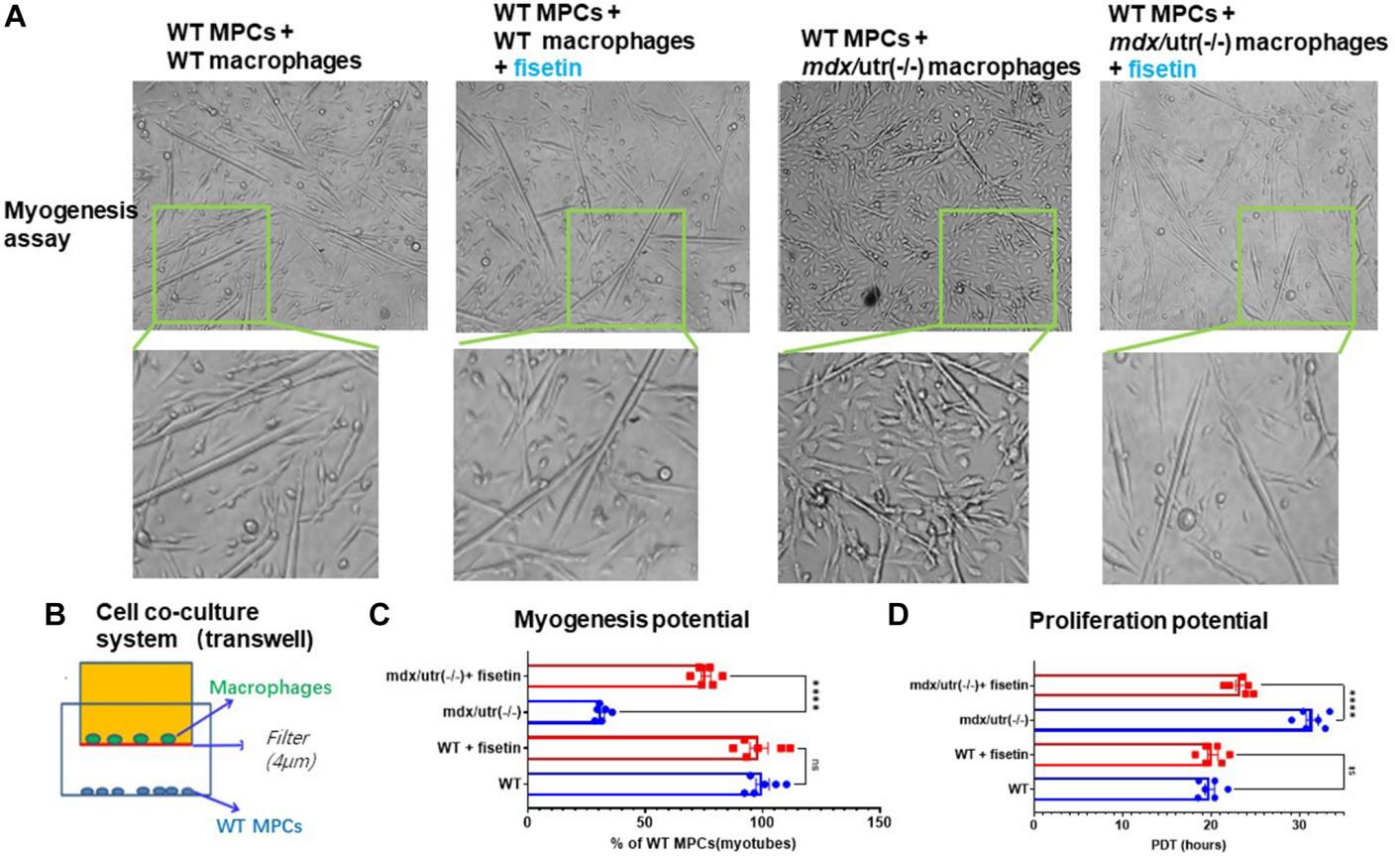
**Macrophages from *mdx*/utro(−/−) muscle impair the function of co-cultured MPCs, which can be rescued with senolytic drug.** (**A**) The progression of myotube formation was tracked in the myogenesis assay of WT MPCs co-cultured with WT or *mdx*/utro(−/−) macrophages, with or without fisetin treatment (20 μM, 96 h). (**B**) Schematic demonstration of the cell co-culture system of WT or *mdx*/utro(−/−) macrophages (upper chamber) and WT MPCs (lower chamber). (**C**) Statistics of result for myogenesis assay. (**D**) Statistics of result for proliferation assay. *n* = 6 (3 cell lineages x 2 replicates/group). Data are presented as mean +/− SD.

A cell coculture system was setup, to verify the potential direct impact of CD68+ macrophages in *mdx*/utro(−/−) muscle may exert on muscle progenitor/stem cells (MPCs). FAPs and MPCs were isolated from muscles of WT and *mdx*/utro(−/−) mice, and CD68+ macrophages from WT or *mdx*/utro(−/−) muscles and WT MPCs were co-cultured in a transwell system, with CD68+ macrophages from WT or *mdx*/utro(−/−) being at the upper chamber and WT MPCs being at lower chamber. Cell myogenesis and proliferation assays of WT MPCs showed that, the WT MPCs co-cultured with CD68+ macrophages from *mdx*/utro(−/−) developed impaired myogenesis and proliferation potentials (Figure 4).

### Treatment of co-culture system of *mdx*/utro(−/−) macrophages and WT MPCs with fisetin rescues the impaired function of WT MPCs

We then examined whether the removal of senescent cells in *mdx*/utro(−/−) macrophages with senolytic drug may rescue the function of muscle stem cells in the cell co-culture system. Fisetin was applied to treat the cell co-culture system of *mdx*/utro(−/−) macrophages and WT MPCs for 48 hr at 20 μM of concentration, and we observed improved myogenesis and proliferation potential of WT MPCs, compared to non-treated controls (Figure 4).

### Treatment of *mdx*/utro(−/−) mice with fisetin results in increased number of muscle stem cells and improved muscle phenotypes

**Figure 5:**
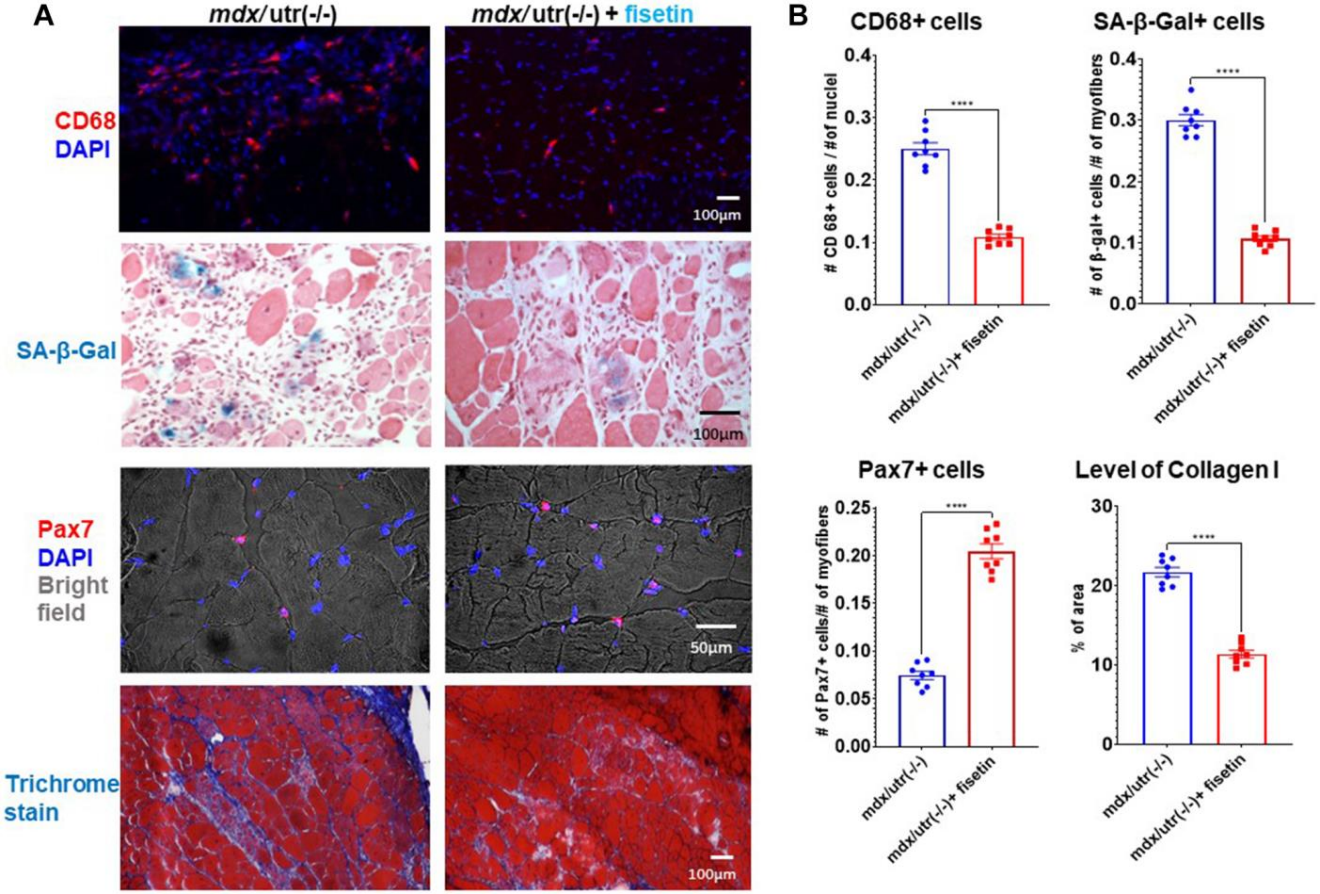
***In vivo* results of fisetin administration in *mdx*/utro(−/−) mice.** (**A**) *mdx*/utro(−/−) mice were treated with fisetin via oral gavage (20 mg/kg for 5 consecutive days each week) for 4 weeks and muscle tissues were analyzed. Immunofluorescent staining of CD68 showed that the number of macrophages was decreased with fisetin administration; staining of SA-β-Gal showed that the number of senescent cells was decreased with fisetin administration; immunofluorescent staining of Pax7 showed that the number of MPCs was increased with fisetin administration; trichrome staining of collage deposition (fibrosis formation) showed that the amount of fibrosis formation was decreased with fisetin administration. (**B**) Statistics of *in vivo* results. *n* = 8 mice/group. Data are presented as mean +/− SD.

Finally, in order to investigate the efficacy of senolytics on muscle health *in vivo*, *mdx*/utro(−/−) mice were treated with fisetin via oral gavage (20 mg/kg for 5 consecutive days each week)) for 4 weeks. Immunostaining of muscle cryosections revealed that, compared to non-treated mice, the number of CD68+ macrophages and SA-β-Gal+ senescent cells was decreased and the number of Pax7+ MPCs was elevated in fisetin treated mice, while muscle phenotypes were also improved by observation of reduced fibrosis formation (collagen deposition) (Figure 5).

## DISCUSSION

Cellular senescence is a defined hallmark of aging, contributing to the aging process and age-related diseases and disorders. Senotherapeutics, including senolytics and senomorphics, has emerged as a promising anti-aging strategy, and was found to be able to prolong healthspan and lifespan of animals [12]. In recent years, senolytics have been widely studied in various types of tissues and disease models and have been proven for their beneficial effect in delaying aging or treating age-related diseases [11–14]. Interestingly, our recent study of *mdx*/utr(−/−) mice revealed that, macrophages are highly prevalent in severely dystrophic muscle of *mdx*/utr(−/−) mice and display senescence-like phenotypes, including the expression of SASP factors and positive staining for C_12_FDG [3]. However, the potential effect and mechanism of senolytics in regulating the function of MPCs in severely dystrophic muscles remains largely unclear. It was recently discovered that the ageing immune system, or immunosenescence, has a causal role in driving systemic ageing and therefore represents a key therapeutic target to delay ageing [5]. Therefore, here we investigated whether senolytic ablation of senescent immune cells in dystrophic muscle of *mdx*/utr(−/−) mice may promote the restoration of exhausted muscle stem cell pool and improve muscle phenotypes. Our current results show that, a substantial fraction of *mdx*/utr(−/−) macrophages develop senescence in the severely dystrophic muscles, which dominantly repress the proper function of MPCs by releasing SASPs; while the elimination of senescent macrophages with fisetin is able to rescue the function of MPCs.

Senescent cells are known as a source of age-related chronic inflammation via secreting multiple pro-inflammatory factors of SASPs [9, 10]. As senescent cells accumulate in the aged tissues, they can exert profound effects on the growth and function of normal cells by releasing SASP factors including exosomes [18, 20]. Senescent cells play a critical role in inducing or mediating various types of aging-related diseases [16–20]. A reduction in the senescent cell burden can be achieved with several different classes of senolytics including the combination of dasatinib and quercetin (D+Q), navitoclax (ABT263) and related Bcl-2 family member inhibitors, HSP90 inhibitors such as 17-DMAG and the flavonoid fisetin [16, 22–27]. Fisetin is a naturally occurring anti-oxidant that scavenges free radicals as well as upregulates synthesis of glutathione, and it also has been reported to inhibit apoptosis-related signaling BCL-XL [26], which may also be important for its senolytic activity. The positive effect of fisetin in removing senescent cell in aged tissues has been well-demonstrated, our current results have further demonstrated that fisetin is effective in removing senescent immune cells in dystrophic muscles.

Senescent macrophages are in fact also found to express senescence-related markers p16(Ink4a) and β-galactosidase (β-gal), and promote inflammation in diseased tissues [25, 26]. Our previous work has indicated increased cellular senescence in dystrophic muscles of *mdx*/utr(−/−) mice [3], however, whether or not macrophages in particular develop cellular senescence and promote senescence associated phenotypes was still unknown. To this end, here we further examined *mdx*/utr(−/−) mice and solved these puzzles.

Immune cells in the skeletal muscle are activated during muscle injury and promote the process of muscle regeneration by coordinating with muscle stem cells. However, studies with severely diseased muscles further demonstrate that immune cells can become dominantly activated and is inductive of increased fatty infiltration and fibrosis formation, while at the same time potently repress the proliferation and function of muscle stem cells [27]. Our current results in severely dystrophic muscle reveal a similar situation of interaction between macrophages and MPCs, showing that the function of MPCs is repressed by the senescent macrophages. As senescent cells accumulate in the aged or diseased tissues, it can exert profound effects on the growth and function of normal cells by releasing SASPs [9, 10]. Fisetin is a naturally-occurring flavone with low toxicity, a selective BCL-XL inhibitor [15], and our current results demonstrate the effective rescuing of muscle stem cell function and dystrophic muscle phenotype with fisetin administration. Because senescent cell usually takes weeks to reaccumulate, intermit administration of senolytics is efficient to achieve ideal results. Here in our study, it shows the weekly administration of fisetin in mice is effective in eliminating many of senescent cells and rescue the growth and function of MPCs.

Still, there is some limitation in our current study, such as the lack of results for other types of senolytic drugs than fisetin in potentially having better senolytic effect and improving severely dystrophic muscle, and results for other types of immune cells than senescent macrophage in potentially impacting the function of muscle stem cells, which can be further studied in our future researches. Also, here we have studied cells from a murine model of DMD, and the potential effect of fisetin in dystrophic muscle cells from human patients is to be further verified.

Therefore, our current results reveal that the senescence of macrophages play an important role in negatively regulating MPC function in severely dystrophic muscles of murine model of DMD, and the elimination of senescent macrophages with senolytics is effective in rescuing MPC function. Our results demonstrate that senescent macrophages may potentially serve as a new therapeutic target for treatment of muscular dystrophy or delaying the aging of skeletal muscle tissues.

## MATERIALS AND METHODS

### Animal models

WT (C57BL/10J, male) mice were obtained from the Jackson Laboratory (Bar Harbor, ME, USA). *Mdx*/utr(−/−) mice (dystrophin/utrophin double knock out, dKO, or *dystrophin*^−/−^:utrophin^−/−^; male mice) were derived from our in-house colony. Mice were housed in groups of 4 on a 12:12-hour light-dark cycle at 20–23°C. At least 8 mice were used in each experimental sample group. All procedures were approved by the Institutional Animal Care and Use Committee (IACUC) at the University of Pittsburgh (IACUC-1109718) and the University of Texas Health Science Center at Houston (AWC-18-0068). All mice were housed and maintained in the Center for Laboratory Animal Medicine and Care (CLAMC) at UTHealth (University of Texas Health Science Center at Houston) in accordance with established guidelines and protocols approved by the UTHealth Animal Welfare Committee.

### Isolation of macrophages from skeletal muscles

Gastrocnemius (GM) muscle tissues were harvested from WT and *mdx*/utr(−/−) mice (8-week old, *n* = 3 for each group), and digested by serial 1-hr incubations at 37°C in 0.2% type XI collagenase (Sigma-Aldrich, Burlington, MA, USA), dispase (grade II, 240 units; Sigma-Aldrich), and 0.1% trypsin (Thermo Fisher Scientific, Waltham, MA, USA), as previously described [6]. Primary cells from muscle tissues were harvested after digestion, and macrophages were then isolated from these primary cells by Fluorescence activated cell sorting (FACS) of CD68+ cells with a cell sorter (BD FACSAria, San Jose, CA, USA). Macrophages were cultured and immunostaining with CD68 antibody was performed to validate the identity of the isolated cells to be macrophages; the primary muscle cells were also immunostained with CD68 antibody to serve as negative control.

### SA-β-gal staining for senescent cells

The presence of senescent cells in skeletal muscle tissue [*n* = 8 for WT or *mdx*/utr(−/−) mice] and cells [*n* = 3 for WT or *mdx*/utr(−/−) cells, with 2 replicates for each cell population] cultured *in vitro* was individually measured using the regular senescence-associated β-Galactosidase (SA-β-gal) Staining Kit (Cell Signaling Technology, Danvers, MA, USA) and CellEvent Senescence Green Detection Kit (fluorescent) (Thermo Fisher Scientific), following the manufacturers’ protocol. The number of cells positive for β-gal activity at pH 6, a known characteristic of senescent cells, was determined.

### Cell co-culture system of FAPs and MPCs

MPCs from WT muscle (*n* = 3, with 2 replicates for each cell population) were seeded in the lower chamber of a transwell (Thermo Fisher Scientific), with macrophage from WT or *mdx*/utr(−/−) mice (*n* = 3, with 2 replicates for each cell population) being seeded in the upper chamber. The pore size of the transwell membrane was 0.4 μm, and coated with a layer of Matrigel (20% of Matrigel in water, 0.2cm of thickness).

### Myogenesis and proliferation assays

For myogenic differentiation assay, WT MPCs (*n* = 3, with 2 replicates for each cell population) were seeded as ~90% confluence in the lower chamber of transwell, with macrophages from WT and *mdx*/utr(−/−) mice (*n* = 3, with 2 replicates for each cell population) being co-cultured in the upper chamber (8000 cells/cm^2^); cells were co-cultured in the DMEM medium containing 2% HS (horse serum), and allowed for differentiation for 96 h with or without fisetin (Selleckchem, Houston, TX, USA), before being fixed and imaged to compare the number of myotubes formed by WT MPCs in different groups (WT MPCs + WT macrophages, WT MPCs + WT macrophages + fisetin, WT MPCs + *mdx*/utr(−/−) macrophages, and WT MPCs + *mdx*/utr(−/−) macrophages + fisetin). The concentration of fisetin used for cells was 20 μM, which was based upon previous studies that reported its senolytic effect [16, 24]. For proliferation assay, WT MPCs were seeded as 2000 cells/cm^2^ in the lower chamber of transwell, with macrophages being co-cultured in the upper chamber (5000 cells/cm^2^); cells were co-cultured in the DMEM medium containing 20% FBS and 1% CEE (Chicken Embryo Extracts) and allowed for proliferation for 72 h with or without fisetin (20 μM) before being observed to compare the number of WT MPCs in different groups and calculate cell population doubling time (PDT).

### Fisetin treatment of cell co-culture system and mice

For *in vitro* cell treatment, fisetin (Selleckchem) was applied to treat the cell co-culture system of *mdx*/utr(−/−) macrophages and WT MPCs (*n* = 3, with 2 replicates for each cell population) at 20 μM of concentration, and the proliferation and myogenesis potential of WT MPCs was then compared to non-treated controls. For *in vivo* treatment of *mdx*/utr(−/−) mice [*n* = 8 for WT or *mdx*/utr(−/−) mice] , fisetin was dissolved in vehicle (10% ethanol, 30% polyethylene glycol, 60% phosal 50 pg). Mice were weighed and given fisetin (20 mg/kg for 5 consecutive days each week) or vehicle control alone by oral gavage for 4 weeks.

### mRNA analysis via reverse transcriptase-PCR

Total RNA was obtained from MPCs or the skeletal muscles of mice (*n* = 8) using the RNeasy Mini Kit (Qiagen, Inc., Valencia, CA, USA) according to the manufacturer’s instructions. Reverse transcription was performed using an iScript cDNA Synthesis Kit (Bio-Rad Laboratories, Inc., Hercules, CA, USA). The sequences of primers are given in Table 1 for SASP genes (CXCL2, MCP1, IL-1α, IL-1β, IL-6 and IFN-γ), p16, IL-10, and GAPDH (glyceraldehyde 3-phosphate dehydrogenase). Real-Time PCR reactions were performed using an iCycler thermal cycler (Bio-Rad Laboratories, Inc.). The cycling parameters used for all primers were as follows: 95°C for 10 minutes; PCR, 40 cycles of 30 seconds at 95°C for denaturation, 1 minute at 54°C for annealing, and 30 seconds at 72°C for extension. All data were normalized to the expression of GAPDH.

**Table 1:** RT-PCR primer sequences.

**Gene**	**Primer sequence**
*GAPDH*	Forward: TCCATGACAACTTTGGCATTG
	Reverse: TCACGCCACAGCTTTCCA
*TNF-α*	Forward: CCTGTAGCCCACGTCGTAG
	Reverse: GGGAGTAGACAAGGTACAACCC
*IL-1α*	Forward: TCTCAGATTCACAACTGTTCGTG
	Reverse: AGAAAATGAGGTCGGTCTCACTA
*IL-1β*	Forward: GCAACTGTTCCTGAACTCAACT
	Reverse: ATCTTTTGGGGTCCGTCAACT
*CXCL1*	Forward: CTGGGATTCACCTCAAGAACATC
	Reverse: CAGGGTCAAGGCAAGCCTC
*MCP1*	Forward: TAAAAACCTGGATCGGAACCAAA
	Reverse: GCATTAGCTTCAGATTTACGGGT
*IFN-γ*	Forward: CAGCAACAGCAAGGCGAAAAAGG
	Reverse: TTTCCGCTTCCTGAGGCTGGAT
*CDKN2A*	Forward: CGCAGGTTCTTGGTCACTGT
	Reverse: TGTTCACGAAAGCCAGAGCG
*IL-10*	Forward: ATTTGAATTCCCTGGGTGAGAAG
	Reverse: CACAGGGGAGAAATCGATGACA

### Trichrome staining

Fibrosis formation in muscle tissues of WT or *mdx*/utr(−/−) mice (*n* = 8) was visualized by Masson trichrome staining with the Trichrome Stain (Masson) Kit (Sigma-Aldrich). Sections were incubated in Weigert’s iron hematoxylin working solution for 10 min and rinsed under running water for 10 min. Slides were transferred to Biebrich scarlet-acid fuchsin solution for 15 min before incubation in aniline blue solution for another 5 min. Slides were then rinsed, dehydrated, and mounted as earlier. The ratio of the area of fibrotic collagen (blue) to the area of normal muscle (red) was quantified to measure fibrosis formation.

### Immunofluorescent staining and imaging

Cultured cells (*n* = 3, with 2 replicates for each cell population) were fixed with 4% paraformaldehyde, and frozen muscle tissue sections were fixed with 10% formalin. The primary antibodies used – CD68 (Abcam, Boston, MA, USA), p-4E-BP1(Cell Signaling), Pax7 (DSHB, Iowa City, IA, USA), Collagen IV (Abcam), and fMHC (fast-type myosin heavy chain, R&D Systems, Minneapolis, MN, USA) – were all applied at a 1:100 to 1:300 dilution. Cell nucleus was stained with DNA binding reagent and 4′,6-diamidino-2-phenylindole (DAPI). Immunofluorescent images of myofibers were imaged and photographed with a Nikon high resolution microscope.

### Measurements of results and statistical analysis

Image analysis was performed using ZEN 2.3 imaging software (ZEISS Microscopy, Germany) and Image J software (version 1.32j; National Institutes of Health, Bethesda, MD, USA). Data from at least six samples from each subject were pooled for statistical analysis. Results are given as the mean ± SEMx. The statistical significance of any difference was calculated using Student’s *t*-test or two-way ANOVA test. *P* values < 0.05 were considered statistically significant.
